# Identification of a Gene Signature for Renal Cell Carcinoma–Associated Fibroblasts Mediating Cancer Progression and Affecting Prognosis

**DOI:** 10.3389/fcell.2020.604627

**Published:** 2021-02-05

**Authors:** Bitian Liu, Xiaonan Chen, Yunhong Zhan, Bin Wu, Shen Pan

**Affiliations:** ^1^Department of Urology, Shengjing Hospital of China Medical University, Shenyang, China; ^2^Department of Radiology, Shengjing Hospital of China Medical University, Shenyang, China

**Keywords:** renal cell carcinoma, cancer-associated fibroblast, weighted gene co-expression network analysis, single sample gene set enrichment analysis, gene signature

## Abstract

**Background:** Cancer-associated fibroblasts (CAFs) are mainly involved in cancer progression and treatment failure. However, the specific signature of CAFs and their related clinicopathological parameters in renal cell carcinoma (RCC) remain unclear. Here, methods to recognize gene signatures were employed to roughly assess the infiltration of CAFs in RCC, based on the data from The Cancer Genome Atlas (TCGA). Weighted Gene Coexpression Network Analysis (WGCNA) was used to cluster transcriptomes and correlate with CAFs to identify the gene signature. Single-cell and cell line sequencing data were used to verify the expression specificity of the gene signature in CAFs. The gene signature was used to evaluate the infiltration of CAFs in each sample, and the clinical significance of each key gene in the gene signature and CAFs was analyzed. We observed that the CAF infiltration was higher in kidney cancer and advanced tumor stage and grade than in normal tissues. The seven key genes of the CAF gene signature identified using WGCNA showed high expression of CAF-related characteristics in the cell clustering landscape and fibroblast cell lines; these genes were found to be associated with extracellular matrix function, collagen synthesis, cell surface interaction, and adhesion. The high CAF infiltration and the key genes were verified from the TCGA and Gene Expression Omnibus data related to the advanced grade, advanced stage, and poor prognosis of RCC. In summary, our findings indicate that the clinically significant gene signature may serve as a potential biomarker of CAFs in RCC, and the infiltration of CAFs is associated with the pathological grade, stage, and prognosis of RCC.

## Introduction

Renal cell carcinoma (RCC) is one of the 10 most deadly cancers globally, causing more than 140,000 deaths each year (Capitanio et al., 2019). Over the past decade, advanced RCC treatment has changed from a nonspecific immune approach to vascular endothelial growth factor targeted therapy, and now to immune checkpoint blockers (Barata and Rini, 2017). Nevertheless, the overall prognosis for patients with advanced RCC remains poor (Bosse and Ong, 2020). Cancer-associated fibroblasts (CAFs) are an important part of the tumor microenvironment (TME), and they can function as tumor promoters and inhibitors (Park et al., 2020). Recently, the benefits of CAFs in tumor progression have been identified (Saini et al., 2020), making them potential targets for the development of novel therapies in the future (Chen and Song, 2019). However, there are very few studies regarding CAFs in RCC.

Previous reports have suggested that CAFs often carry recognized markers for identification, such as α-smooth muscle actin (αSMA) and fibroblast activation protein α (FAPα) (Nurmik et al., 2020). There are specific CAF genes in different cancer tissues; thus, research using these recognized markers as gene signatures may cause deviations in the evaluation of CAFs in the TME. Single-cell transcriptomes can reveal cell specificity in specific tumor tissues, such as the cellular identity of human kidney tumors, which contain fibroblasts (Young et al., 2018). Although single-cell sequencing technology can classify cells and identify specific markers, the number of cells measured and the source of cases are limited, which may lead to bias.

The R package Weighted Gene Coexpression Network Analysis (WGCNA), which has been widely used recently (Pan et al., 2019a), has the potential to recognize CAF-specific markers (Liu et al., 2021). In the TME, different cells have varying specific gene expression, and the fluctuation of cell class proportions affects the expression of their specific genes. However, the expression of highly specific genes is less disturbed by the proportion of other cells. These genes show a strong correlation that cannot be offset as the fraction of cells changes. WGCNA can make good use of the special properties of the TME to identify cell-specific gene sets in a large number of heterogeneous samples.

In our study, we evaluated the infiltration status of the stroma and CAFs using previously identified gene signatures using the R packages: Estimation of STromal and Immune cells in MAlignant Tumor tissues with Expression data (ESTIMATE) and Estimate the Proportion of Immune and Cancer cells (EPIC). By correlating the fraction of CAFs with the gene module calculated by WGCNA, the specific CAF gene set can be found. By verifying the single-cell and cell line sequencing data, we proved the expression specificity of the gene signature of the kidney renal clear cell carcinoma (KIRC) CAFs. Finally, we verified the clinical significance of gene signature and CAFs in KIRC, the main pathological type of RCC.

## Materials and Methods

### Data Download and Processing

RNA-seq and related clinical data for human KIRC samples were obtained from The Cancer Genome Atlas (TCGA) database (portal.gdc.cancer.gov), containing 611 tissues and 530 cases. These data were updated on April 10, 2020. RNA-seq data of 72 normal and 539 cancer samples were combined into matrix files.

A previous study using single-cell transcriptomes revealed the cell-specific genes of different cells in RCC and summarized the markers of canonical cell types known in the existing literature (Young et al., 2018). To explore whether the gene signatures screened by WGCNA are consistent with the cell-specific genes revealed by single-cell transcriptomes, we downloaded and analyzed the single-cell RNA (scRNA) sequencing data from renal tumors (Young et al., 2018). Based on the Seurat R package and Scanpy python package, we processed the scRNA sequencing data and generated cell clustering FeaturePlot and TracksPlot. FeaturePlot was used for Uniform Manifold Approximation and Projection (UMAP) for dimension reduction.

To further prove the expression specificity of gene signatures in fibroblasts, we downloaded gene expression data of cell lines from the depmap portal (depmap.org/portal) containing 1,270 cell lines and 19,144 genes. According to the tumor environment of kidney cancer, we selected cell lines labeled as blood, fibroblast, kidney, lymphocyte, and plasma cells as the comparison objects. We further obtained the transcriptome count data of the cell lines from the Cancer Cell Line Encyclopedia (CCLE), and the data version date is September 29, 2018. We extracted RNA-seq data of 32 kidney cancer and 37 fibroblast cell lines and used the R 3.6.2 software to generate heatmap and volcano maps to show the differentially expressed genes (DEGs).

To verify the clinical significance, series GSE29609 containing various TNM-stage samples from 39 patients with survival information, series GSE53757 containing 101 normal tissue-tumor pair samples, and series GSE73731 containing 265 tumor samples were downloaded from the Gene Expression Omnibus (GEO) database.

### Stromal and Immune Components

Stromal and immune fractions were evaluated by ESTIMATE (Yoshihara et al., 2013). Using the ESTIMATE R package in R 3.6.2, tumor stromal and immune cell infiltrations of TCGA-KIRC samples were calculated from the profiles of two gene sets, including 141 genes. The preliminary calculated stromal and immune scores are used to observe the differences between stage and grade.

### Fractions of Various Cells in the TME

In order to continue to explore the changes in matrix components in tumor tissues, EPIC (gfellerlab.shinyapps.io/EPIC_1-1/) was used to estimate the fraction of CAFs. The EPIC application is designed to estimate the proportion of immune and cancer cells from the bulk tumor gene expression data (Racle et al., 2017). This is done by fitting gene expression reference profiles from the main nonmalignant cell types, simultaneously accounting for an uncharacterized cell type without prior knowledge about it. EPIC establishes reference gene expression profiles for major tumor-invasive immune cell types (CD4^+^ T, CD8^+^ T, B, natural killer, and macrophages) and further deduces the reference spectra of CAFs and endothelial cells.

### WGCNA

According to the principle of WGCNA calculation, highly coexpressed gene modules recognized by WGCNA can be considered as a set of specifically expressed genes of a certain type of cells in tumor tissues. More tissue samples and greater fluctuations in the composition ratio make this method more suitable for identifying cell-specific expression gene sets. In a complex tumor environment, the detection of cell-specific expression genes is mainly determined by the proportion of one cell type, whereas other cells will have less interference with such genes.

### Highly Coexpressed Gene Set–Gene Module

The WGCNA package in R was used to perform a weighted correlation network analysis (Langfelder and Horvath, 2008). To exclude highly correlated genes with no significant changes in expression, the genes with a high variance of 25% were selected (Pan et al., 2019a,b). After filtering the RNA-seq data to remove the outliers, we constructed a Pearson correlation matrix and generated a weighted adjacency matrix emphasizing strong correlations and penalizing weak correlations. After selecting an appropriate β value via power calculation, a topological overlap matrix (TOM) was produced (Botia et al., 2017). Based on TOM-dependent dissimilarity measurements, average-linkage hierarchical clustering and module dendrograms were used to construct modules with a minimum gene dendrogram size of 30.

### Identification of Interested Modules

Gene significance (GS) was calculated to measure the correlation between genes and cell fractions and determine the significance of each module. The expression patterns of all module eigengenes were summarized as a single feature within a given module (Pan et al., 2019b). We selected a cutoff threshold of <0.25 to merge some modules with similar heights and increase module capacity (Pan et al., 2019a).

### Representative Genes in a Module

GS and module membership (MM, the correlation between the genes and gene expression profiles of a module) can be used to assess the gene–phenotype relationships and their importance in the modules. Similar to most previous studies, we defined high MM and GS values (MM.cor and GS.cor, respectively) as the threshold to identify representative genes in a module (Pan et al., 2019a).

### CAF-Specific Markers

The markers of CAFs have been collated and summarized (Lennon et al., 2014; Gascard and Tlsty, 2016; Yu et al., 2019). They comprise specific as well as nonspecific markers of CAFs. Here, we carried out correlation analysis using TCGA data to prove the reliability of the signature of CAFs in KIRC.

### Pathway and Process Functional Enrichment Analysis

To further verify the function of genes, a Metascape (metascape.org) search was performed for gene enrichment analysis. The Kyoto Encyclopedia of Genes and Genomes, Gene Ontology, Reactome gene sets, and CORUM provide ontology sources for pathway and process enrichment analysis (Zhou et al., 2019). Terms with *p* < 0.01, a minimum count of 3, and an enrichment factor > 1.5 were collected and grouped into clusters based on their similarities. Subsets of enriched terms with a similarity score > 0.3 were connected by the edges to render a network plot and further capture the relationships between the terms.

### Analysis of the Clinical Significance of Each Gene in the Gene Signature

To explore the clinical significance of each gene in the KIRC CAF signature, we performed an overall survival analysis using the median method and the best triple groups selected with the X-tile tool (Camp et al., 2004). A receiver operating characteristic (ROC) curve analysis was used to verify the diagnostic efficacy of the gene signature in the high-stage-grade and low-stage-grade groups.

### Protein Expression of the Gene Signature

To explore the expression of key genes in kidney adenocarcinoma and stromal cells, we obtained immunohistochemistry (IHC) images of KIRC from The Human Protein Atlas (http://www.proteinatlas.org), which aims to map human proteins in pathology (Uhlen et al., 2017), and selected the images containing both the cells with the assistance of expert pathologists. We also used the markers fibronectin 1 (FN1) and FAP of CAFs and desmin (DES), a smooth muscle cell marker, to confirm the position and approximate shape of the stromal part and exclude muscle tissues (Gascard and Tlsty, 2016; Lee et al., 2020; Liu et al., 2021).

### Single Sample Gene Set Enrichment Analysis

Single-sample gene set enrichment analysis (ssGSEA) can identify and distinguish the changes in a class of cells in different samples based on gene signatures (Zhang et al., 2018; Pan et al., 2019b). We used the GSEA program to obtain the absolute enrichment scores from the CAF gene signature previously identified using the WGCNA. The infiltration level of one cell type was quantified by ssGSEA in the R package gsva, where ssGSEA utilized a deconvolution approach including myofibroblasts and fibroblasts.

### Statistical Analysis

GraphPad Prism 8.4 (GraphPad Software, San Diego, CA, USA) was used for statistical analysis. Statistical significance was determined using the Student *t* test (two-tailed) for two groups, one-way analysis of variance, and/or Tukey test for more than two groups. Pearson χ^2^ test or Fisher exact test was used to analyze the correlation between fibroblasts and clinicopathological parameters. Kaplan–Meier log-rank test was used to calculate the association of CAFs with overall survival. Cox proportional hazards regression model was used to calculate the association of the expression levels of genes with overall survival. Each group of data is presented as mean ± SD. In the bar graphs of figures, ^*^p < 0.05, ^**^p < 0.01, ^***^p < 0.001, and ^****^p < 0.0001, respectively. *p* < 0.05 was considered to indicate a statistically significant difference.

## Results

### Changing Trends in the Stroma and CAFs

**Figure 1:**
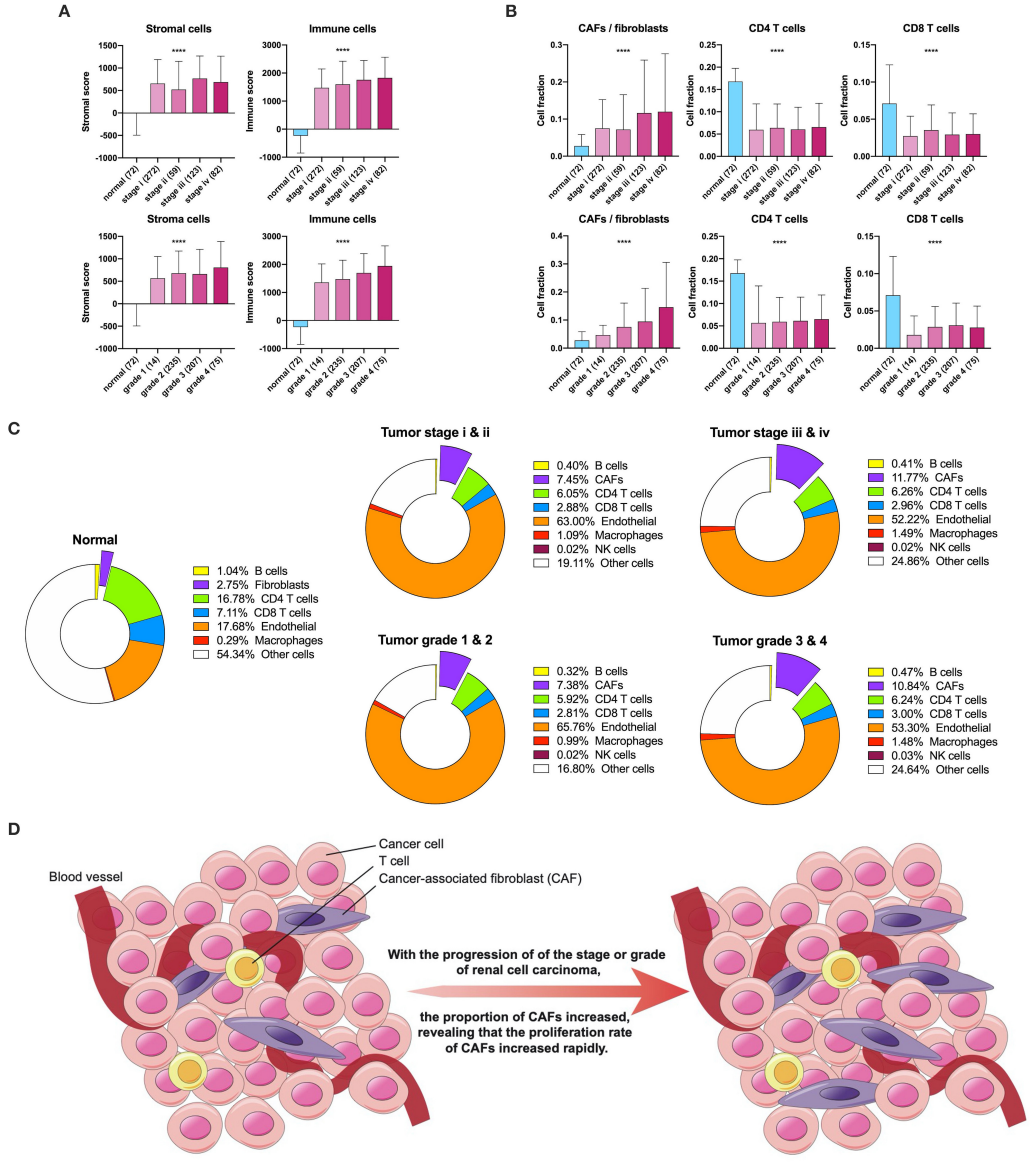
Stroma and CAFs in KIRC. **(A)** Stromal and immune scores in various stages and grades. **(B)** CAFs, CD4, and CD8 T cells in various stages and grades. **(C)** Cell fractions of fibroblasts or CAFs differ in tissue types, stages, and grades. **(D)** Increased proliferation rate of CAFs during tumor progression. ^****^p < 0.0001.

First, we used ESTIMATE and EPIC to approximately calculate the infiltration of the stroma, CAFs, and immune cells in KIRC. The ESTIMATE result found that tumor tissues had a higher level of stromal and immune infiltration than normal tissues (Figure 1A). In EPIC, the proportion of CAFs showed a tendency to increase with tumor stage and grade progression, but the proportion of CD4^+^ and CD8^+^ T cells had no significant change in KIRC (Figure 1B). To visualize the scale changes in all cells in EPIC, we show the proportion of CAFs with the progression of stage and grade using the average fraction pie chart (Figure 1C). Based on the above results, the proportion of CAFs increases during tumor progression or can be considered as an increase in the rate of proliferation of CAFs (Figure 1D). Because the CAF fraction of EPIC is estimated based on gene signatures from other tissues, we suspected that this fraction may contain different cells and defined them as rough CAFs.

### Identification of Gene Modules and Gene Signature Correlated to CAFs

**Figure 2:**
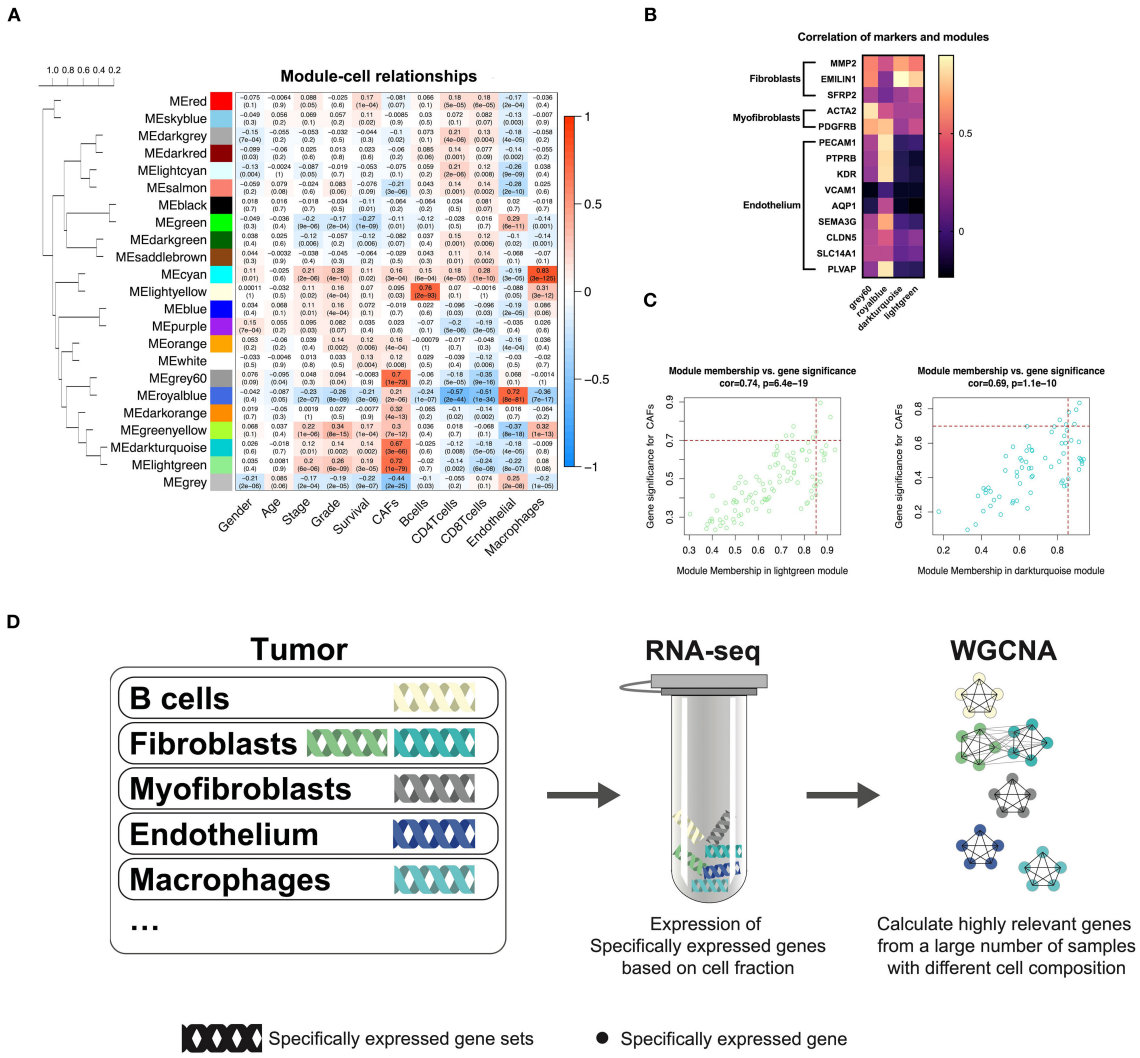
WGCNA recognizes cell-specific gene modules. **(A)** Correlation between the gene modules and traits, including clinical parameters and cell fractions of various cells estimated by EPIC. Correlation coefficients and P values are presented in each cell. The dendrogram on the left shows the degree of difference between the modules. **(B)** MM.cor calculated by WGCNA of canonical markers and grey60, royal blue, dark turquoise, and light green modules. **(C)** Genes with MM.cor > 0.85 and GS.cor > 0.7 were defined as specific markers for CAFs in dark turquoise and light green modules. **(D)** Cell-specific genes can be recognized by WGCNA as a highly coexpressed gene set.

TCGA-KIRC sequencing samples provided WGCNA with 530 cases to effectively and objectively identify cell-specific gene sets. We chose β = 6 (no scale *R*^2^ = 0.814) as a soft threshold to construct a scale-free network. Three modules correlated with the CAF fraction of approximately 0.7. Among them, dark turquoise and light green had a very close relationship in the cluster tree, which could originate from the identical type of cells; however, the grey60 module was not homologous to them (Figure 2A). According to the canonical cell types in normal human kidney tissue, except for the epithelial cells of different microanatomical regions and immune cells, the remaining cells are mainly vascular endothelial cells, fibroblasts, and myofibroblasts (Young et al., 2018). We compared the endothelium, fibroblast, and myofibroblast markers (Young et al., 2018), with MM.cor of different modules. The results show that the dark turquoise and light green modules are closely related to fibroblasts and that the grey60 and royal blue modules are closely related to myofibroblasts and endothelial cells, respectively (Figure 2B). According to the fibroblast modules, dark turquoise and light green, we defined the gene signature or key genes of KIRC CAFs under the conditions of GS.cor > 0.70 and MM.cor > 0.85 (Figure 2C). KIRC CAF-specific gene signature contains seven genes, collagen type i alpha 1 chain (*COL1A1*), collagen type I alpha 1 chain (*COL1A2*), collagen type V alpha 1 chain (*COL5A1*), collagen type XVI alpha 1 chain (*COL16A1*), elastin microfibril interfacer 1 (*EMILIN1*), lysyl oxidase like 1 (*LOXL1*), and lumican (*LUM*).

### WGCNA Can Sort Cell-Specific Expressed Genes

In previous studies, we have demonstrated that WGCNA can recognize genes specifically expressed by CAFs in bladder cancer (Liu et al., 2021). Based on the same calculation principle, WGCNA can also identify the cell-specific expression gene set in KIRC. However, not all cells have cell-specific gene sets, illustrated by the WGCNA KIRC results. We identified only five specific gene modules for cells (Figure 2D). CAFs contain two kinds of cells, myofibroblasts, and fibroblasts. According to the clustering relationship of modules shown in Figure 2A and the correlation between markers and modules shown in Figure 2B, the grey60 module is a representative gene set of myofibroblasts, and the dark turquoise and light green modules are representative gene sets of fibroblasts. The WGCNA calculation principle is to identify highly coexpressed gene sets, while the expression of cell-specific genes changes according to the change in the proportion of cells. Although other cells may interfere with these gene expressions, the impact on their highly coexpressed relationship is limited. We have drawn a schematic diagram to show this WGCNA function (Figure 2D). Obtaining cell-specific gene sets relies on a large number of samples with high tumor heterogeneity and cells with specific expression genes.

### Distribution and Expression of the Key Genes in the scRNA Sequencing

**Figure 3:**
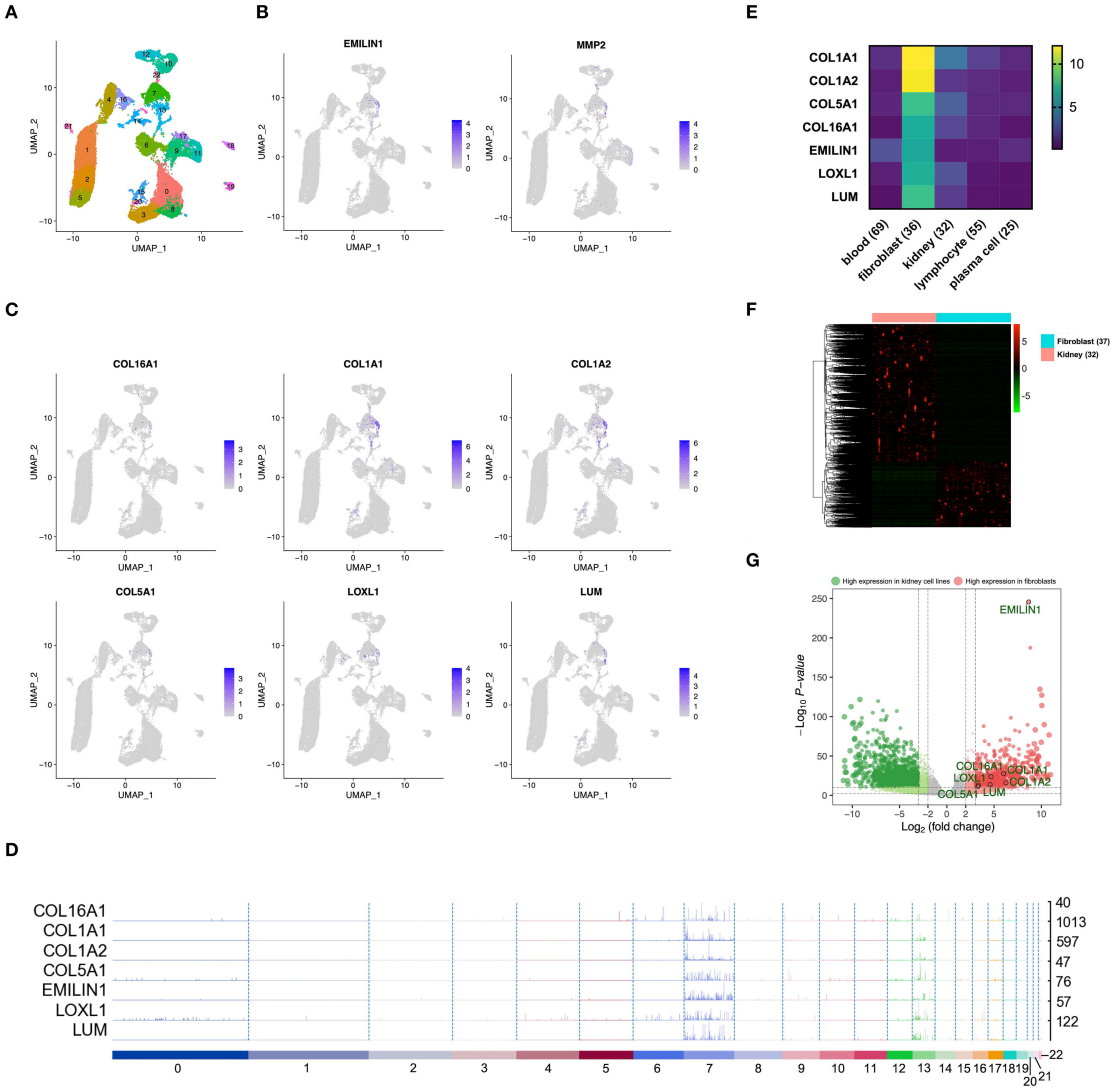
The identified KIRC CAF gene signature is specifically expressed in CAFs. **(A)** Cell cluster feature plot constructed from renal tumor single-cell sequencing data. **(B)** Fibroblast markers EMILIN1 and MMP2 are highly expressed in some cells, mainly in cell cluster 7. **(C)** In addition to EMILIN1, the other six key genes are also highly expressed in some cells in cell cluster 7. **(D)** Trackplot shows that the seven key genes are mainly highly expressed in cell cluster 7. These genes are also expressed in cell cluster 13 because the two groups of cells very similar and are located very close in the landscape. **(E)** Among the different cells in the renal cancer microenvironment, the seven key genes all have the highest expression in fibroblasts. **(F)** The heatmap shows the differential genes between the fibroblast cell lines and the renal cancer cell lines. The threshold of the differential gene is set to |fold change|> 2 and p < 0.01. **(G)** The volcano map shows the differential genes between the kidney cancer cell lines and the fibroblast cell lines. The seven key genes are marked by black circles.

In order to confirm whether WGCNA can identify KIRC CAF-specific genes, we observed the distribution and expression of key genes in the cell landscape based on the scRNA sequencing of renal tumors. According to the UMAP dimensionality reduction algorithm, we used two-dimensional plots to display the expression distribution of cells and used the same color to label the cells in the same cluster (Figure 3A). In the 23 cell clusters, we used the validated markers *EMILIN1* and matrix metallopeptidase 2 (*MMP2*) to label fibroblasts in cell cluster 7 (Figure 3B) (Young et al., 2018). In addition to *EMILIN1*, the other six KIRC CAF-specific genes also showed consistently high expression in cell cluster 7 in the cell FeaturePlot (Figure 3C). Moreover, the TracksPlot showed that the seven key genes have high expression in cell cluster 7 (Figure 3D). Because of the low expression of collagen in other cells, changes in the ratio of fibroblasts can significantly affect collagen expression in tissues.

### Expression of the Key Genes in Cell Lines

To verify the expression specificity of the seven key genes, we compared the expression levels of the genes in the cell lines existing in the renal tumor environment. According to the cells contained in the KIRC tissue, we used the cell line classification in the CCLE to select blood, fibroblast, kidney, lymphocyte, and plasma cell lines that exist in the same TME to compare the gene expression levels. The expression of the seven key genes in fibroblasts was much higher than that in the other cells (Figure 3E). We further applied the data of kidney cancer and fibroblast cell lines in CCLE, using |fold change| >2 and *p* < 0.01 as the threshold to identify the DEGs between the 2 types of cells (Figure 3F). The volcano map revealed that the key genes show higher expression in fibroblasts than in kidney cancer cells (Figure 3G).

### Correlation Between the Key Genes and CAF Markers

**Figure 4:**
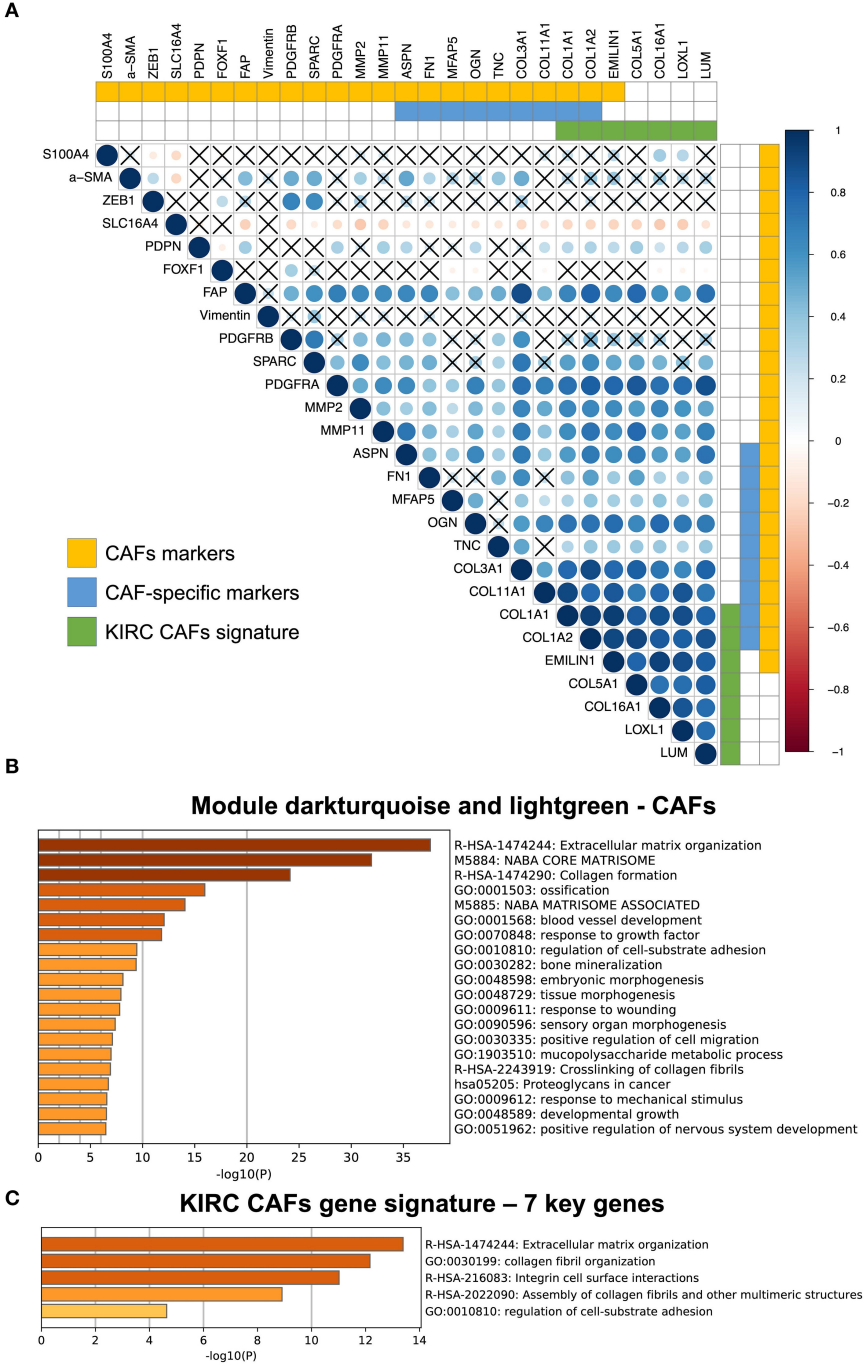
Correlation between KIRC CAF gene signature and CAF-specific markers, and functional analysis. **(A)** Correlation between KIRC CAF signature and CAF markers. “X” represents FDR ≥ 0.05. **(B)** Functional enrichment analysis of dark turquoise and light green modules. **(C)** Functional enrichment analysis of KIRC CAF gene signature.

Among the CAF markers and CAF-specific markers, there were three and two intersections with the signature of CAFs in KIRC, respectively. The seven key genes showed very high coexpression (Figure 4A). CAF-specific markers showed a significantly higher correlation with the signature than did the nonspecific markers.

### Functions of the KIRC CAF Gene Signature

After analyzing and identifying cell-specific genes, we used Metascape (metascape.org) to further verify the functions of these specific genes. First, we performed a functional analysis of the specific modules, dark turquoise and light green, of CAFs, as they are closely related to the extracellular matrix (Figure 4B). Next, a functional analysis of the gene signature containing the seven key genes also showed that these genes were highly associated with extracellular matrix function, as well as collagen synthesis, cell surface interaction, and adhesion (Figure 4C).

### Clinical Significance of the Key Genes

**Figure 5:**
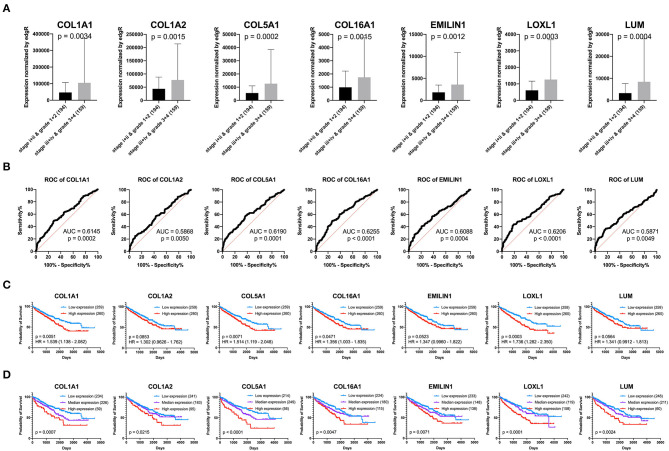
Clinical significance of seven key genes. **(A)** The expression of key genes in different pathological grades and stages. **(B)** ROC curve analysis. The diagnostic efficacy of the nonprogressive group and progressive group was mostly >0.6. **(C)** Grouped survival analysis by the median. **(D)** Survival analysis of each key gene using the best out-based cut-point calculated by X-tile. The higher the expression of all key genes, the worse the prognosis.

Previous results have suggested that the proportion of CAFs in pathological tissues in KIRC increases with the progression of tumors (Figure 1D), and the dark turquoise and light green modules representing CAFs are significantly related to stage, grade, and survival in KIRC (Figure 2A). Moreover, the relationship between the seven genes of the KIRC CAF gene signature and clinicopathological parameters and prognosis was also significant. We defined the progressive group as stages I and II and grades 1 and 2, and the nonprogressive group as stages III and IV and grades 3 and 4. The progressive group had higher key gene expression compared to the nonprogressive group (Figure 5A). In the ROC analysis, the area under the curve of the progressive and nonprogressive tumors was mostly >0.6 (Figure 5B). In the survival analysis, all the seven key genes in the median grouping method showed that the prognosis of the high expression group was worse than that of the low expression group (Figure 5C). In the survival analysis divided into three groups with the assistance of X-tile, all the key genes showed significant prognostic values (Figure 5D). In the univariate analysis, the seven key genes showed remarkable prognostic significance in all 530 TCGA-KIRC cases. According to the multivariate analysis, only LOXL1 had a prognostic value (Table 1).

**Table 1:** Univariate and multivariate analyses of CAFs gene signature genes with overall survival in TCGA KIRC cohort.

	**Univariate analysis**		**Multivariate analysis**	
	**HR (95% CI)**	***P* value**	**HR (95% CI)**	***P* value**
**TCGA KIRC (*****n*** **=** **530)**				
COL1A1 (≥median vs. < median)	1.398 (1.128–1.733)	0.002	1.273 (0.81–2)	0.295
COL1A2 (≥median vs. < median)	1.24 (1.003–1.532)	0.047	0.718 (0.467–1.103)	0.13
COL5A1 (≥median vs. < median)	1.377 (1.112–1.706)	0.003	1.323 (0.846–2.068)	0.22
COL16A1 (≥median vs. < median)	1.256 (1.016–1.553)	0.035	1.005 (0.716–1.412)	0.976
EMILIN1 (≥median vs. < median)	1.256 (1.016–1.552)	0.035	0.93 (0.636–1.358)	0.707
LOXL1 (≥median vs. < median)	1.482 (1.195–1.837)	<0.001	1.397 (1.053–1.853)	0.02
LUM (≥median vs. < median)	1.271 (1.028–1.572)	0.027	0.99 (0.707–1.385)	0.952

**Figure 6:**
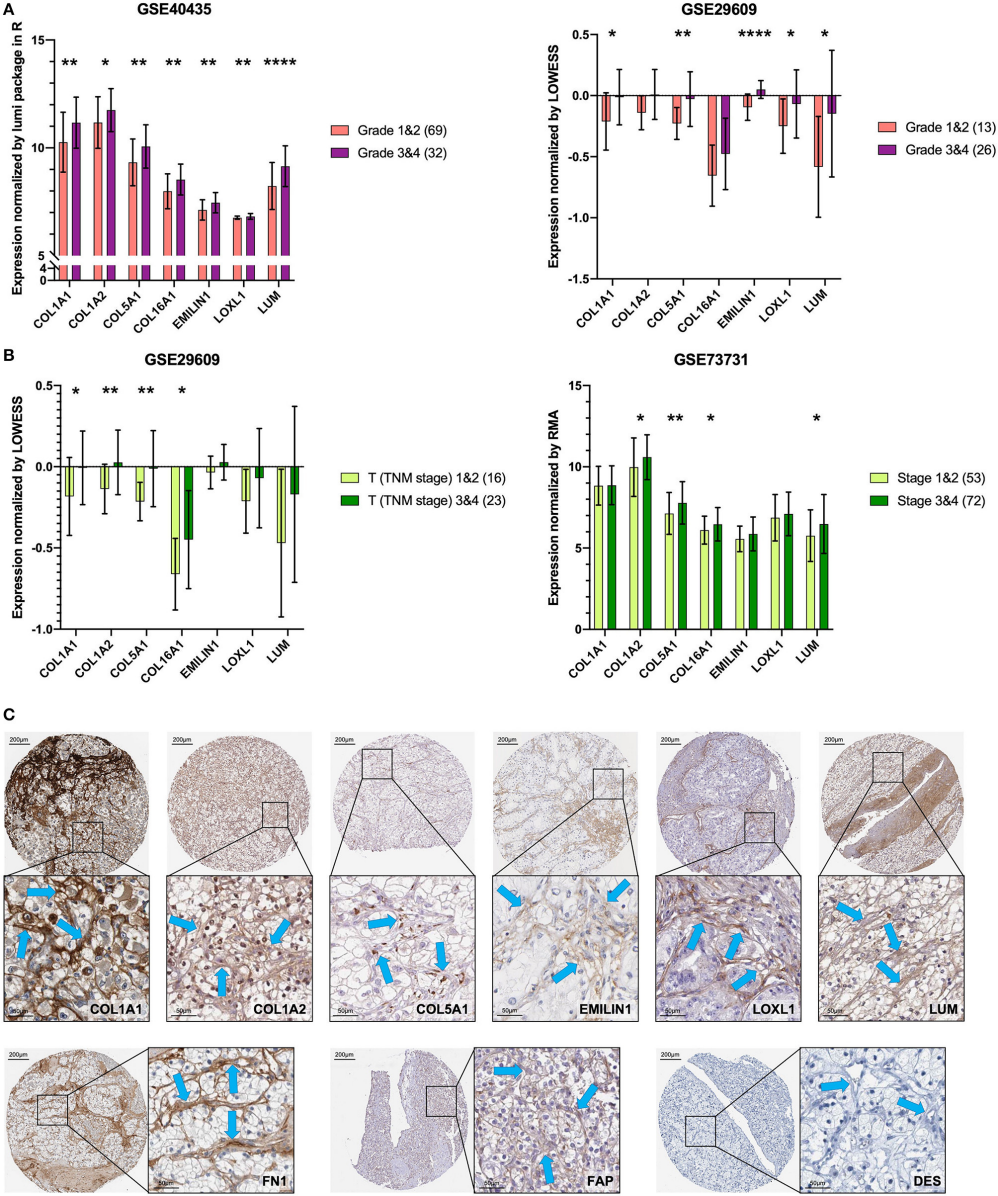
Key genes are specific markers of CAFs in KIRC. **(A)** The key genes are more highly expressed in grades 3 and 4. **(B)** The key genes are more highly expressed in T stages 3 and 4 and stages 3 and 4. **(C)** The expression of the key genes, FN1 and FAP was higher in stromal cells than in kidney cancer cells. The expression of DES was not detected in stromal cells and kidney cancer cells. The blue arrow indicates the stromal part. ^*^p < 0.05, ^**^p < 0.01, and ^****^p < 0.0001.

To verify the external data, we identified seven key genes with significantly high expression in grades 3 and 4 groups in GSE40435 and a trend of high expression in grades 3 and 4 groups in GSE29609 (Figure 6A). In the GSE73731 stage or GSE29609 T stage analysis, all the key genes were overexpressed in the relatively advanced renal tumors, and the expression of more than half of the genes was statistically significant (Figure 6B).

### Proteins of the Key Genes in Pathology

The results of previous analyses are based on transcriptome data. In order to further explore the protein expression location of the key gene, we used IHC images for preliminary analysis. Except for *COL16A1*, which does not have the pathological IHC of renal adenocarcinoma, the other six key gene proteins showed consistent CAFs-containing stromal high expression characteristics in IHC of renal adenocarcinoma (Figure 6C). The CAFs-containing stroma without DES staining had higher FN1 and FAP staining than did the renal cancer cells (Figure 6C).

### CAFs and Related Clinicopathological Parameters

**Figure 7:**
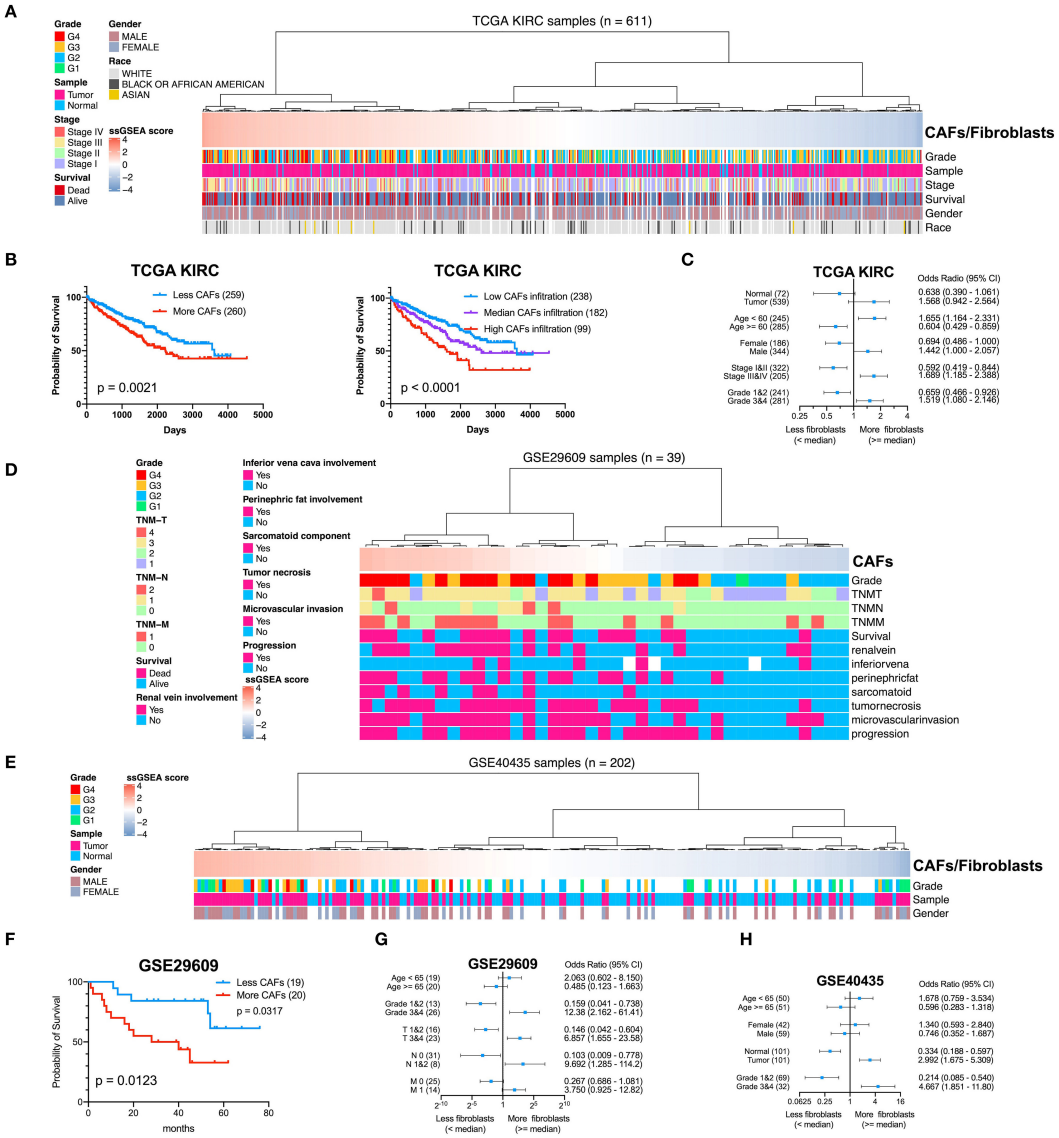
Clinical significance of CAFs. **(A)** Comparison of the infiltration level of CAFs or fibroblasts in each sample in KIRC according to gene signature. **(B)** Survival analysis shows that high CAF infiltration correlates with poor prognosis. **(C)** χ^2^ test reveals the relationship between CAFs or fibroblasts and clinicopathological parameters in KIRC. **(D)** The CAF infiltration level of each sample in series GSE29609. **(E)** The CAF infiltration level of each sample in series GSE40435. **(F)** In GSE29609, patients with higher fibroblast infiltration had a worse prognosis. **(G)** Fisher exact test reveals a relationship between CAFs and clinicopathological parameters in GSE29609. **(H)** χ^2^ test reveals the relationship between CAFs or fibroblasts and clinicopathological parameters in GSE40435.

To determine the clinical significance of CAFs, we used the seven genes of the KIRC CAF gene signature to calculate the infiltration score of CAFs for each sample through ssGSEA. Moreover, we used the ssGSEA score to calculate the infiltration level of CAFs or fibroblasts in 611 samples of TCGA (Figure 7A). By median grouping and triple grouping in 519 cases with tumor stage and survival clinical information as available, the patients with high CAF infiltration showed poor prognosis (Figure 7B). The ssGSEA score of CAFs or fibroblasts was grouped according to the median of the corresponding cases of different clinicopathological parameters. Increased fibroblast infiltration is related to age <60 years, male, stages III and IV, and grades 3 and 4 parameters (Figure 7C). Moreover, in the data verification of series GSE29609 and GSE40435, we used the same gene signatures to score CAF or fibroblast infiltration (Figures 7D,E). In GSE29609, highly infiltrating fibroblasts were related to a worse prognosis (Figure 7F). In the univariate analysis, although only COL1A1 and EMILIN1 have significant prognostic significance, which was applicable for only a few cases, the hazard ratio of all genes was >1 (Table 2). Regarding clinicopathological parameters, a higher fibroblast count is associated with grades 3 and 4, T stages 3 and 4, and N stages 1 and 2 (Figure 7G). In GSE40435, a higher fibroblast count is associated with grades 3 and 4 and tumor tissue (Figure 7H). Although age and fibroblast infiltration levels did not show significant differences, the odds ratio value shows that their trend is consistent, with age <60 or 65 years related to high CAF infiltration.

**Table 2:** Univariate and multivariate analyses of CAFs gene signature genes with overall survival in GSE29609 cohort.

	**Univariate analysis**		**Multivariate analysis**	
	**HR (95% CI)**	***P* value**	**HR (95% CI)**	***P* value**
**GSE29609 (*****n*** **=** **39)**				
COL1A1 (≥median vs. < median)	2.108 (1.03–4.315)	0.041	1.798 (0.854–3.782)	0.122
COL1A2 (≥median vs. < median)	1.462 (0.734–2.91)	0.28		
COL5A1 (≥median vs. < median)	1.976 (0.972–4.019)	0.06		
COL16A1 (≥median vs. < median)	1.652 (0.816–3.346)	0.163		
EMILIN1 (≥median vs. < median)	2.102 (1.002–4.412)	0.049	1.788 (0.829–3.858)	0.138
LOXL1 (≥median vs. < median)	1.924 (0.938–3.947)	0.074		
LUM (≥median vs. < median)	1.885 (0.925–3.843)	0.081		

## Discussion

A prognosis of advanced renal cancer is difficult to be significantly improved by drug treatment (Liu et al., 2018; Yang et al., 2019). The mechanism of renal cancer progression is still unclear; however, the role of TME in KIRC is gaining attention (Vuong et al., 2019). CAFs are the main components of TME; they exchange signals with cancer cells during tumor progression, leading to a variety of treatment failures (Errarte et al., 2020). In our work, we revealed that high CAF infiltration in KIRC is related to poor prognosis and advanced pathological grade and stage. The CAF-specific gene signature of KIRC recognized by WGCNA was clinically significant. With the progression of tumor pathological grade and stage, the infiltration level of CAFs increased significantly. KIRC-specific CAF gene signature not only can provide help for personalized research and treatment of KIRC but may also serve as an essential basis for future treatment of CAFs in KIRC.

It is vital to accurately define CAF markers within KIRC. For CAFs related to tumor progression and prognosis in KIRC, we identified specific markers of CAFs based on WGCNA with advantageous characteristics. Compared with the previous CAF infiltration level calculated based on universal markers, our KIRC CAF-specific markers are more tumor-specific, and the calculation of KIRC CAF infiltration based on the transcriptome can be more accurate. The definition of KIRC CAF-specific markers will provide more foundation and help for subsequent research, such as the sorting of scRNAs, cell positioning, and targeted cell therapy. In short, it is meaningful to accurately define the markers of a class of cells related to staging and prognosis in RCC.

The standard treatment of many advanced cancers, including RCC, has changed from nonspecific immunotherapy or chemotherapy to targeted therapy and immune checkpoint block therapy, from a single treatment to a combined treatment, and from a unified treatment to a personal treatment (Gotwals et al., 2017). The combined precise treatment of cancer and stroma should be the future treatment strategy, which may greatly improve patient prognosis (Valkenburg et al., 2018). The TME and heterogeneity within the tumor provide invasive cells with advantageous conditions for cloning and growth (Parker et al., 2020). In our research, we have shown that fibroblasts are potential invasive cells in the stroma of RCC.

CAFs affect cancer patients' prognosis and are related to treatment resistance (Paulsson and Micke, 2014). The interaction between tumor cells and CAFs may cause treatment failure (Errarte et al., 2020). CAFs promote tumor matrix deposition and remodeling in the TME (Sahai et al., 2020), help cancer cells evade immune surveillance (De Jaeghere et al., 2019), and achieve resistance to immunotherapy (Galvani et al., 2020). A study showed that the extracellular matrix could reduce the effectiveness of immune checkpoint blockers (Wang et al., 2018). Because of the limited understanding of the origin and function of CAFs, it will be a challenge to target them in the future (Sahai et al., 2020).

It is imperative to accurately define the specific markers of fibroblasts in various cancers, not only to identify fibroblasts but also to accurately assess their infiltration level. Previous cancer studies have always confirmed fibroblasts based on the recognition of FAP and αSMA (Wu et al., 2020). However, this only constitutes a basic distinction from cancer cells, and CAFs cannot be further classified. For example, in kidney single-cell sequencing, *EMILIN1, MMP2*, and secreted frizzled related protein 2 (*SFRP2*) are used to sort fibroblasts, whereas αSMA and *PDGFRB* are used to sort myofibroblasts (Young et al., 2018). In the TME of different cancers, fibroblasts also have different specific expression markers from the other surrounding cells. Therefore, identifying cancer-specific CAF markers as targets is a goal for precise treatment or diagnosis.

With regard to CAFs, there is no definite classification of related cell subgroups. At present, CAFs mainly include fibroblasts and myofibroblasts, which can be distinguished based on phenotype and related markers (Young et al., 2018). In this study, we defined fibroblasts as CAFs excluding myofibroblasts. Myofibroblasts, considered to represent an activated fibroblast phenotype, were originally identified as the cells responsible for wound contraction. Myofibroblasts are large cells with long processes resembling fibroblasts, commonly identified by the expression of αSMA. Therefore, myofibroblasts have attracted much attention in renal fibrosis (Strutz and Zeisberg, 2006); however, research regarding the role of myofibroblasts in RCC is still relatively limited.

WGCNA can even outperform single-cell sequencing in identifying cell-specific markers. First, the number of samples that WGCNA can process ranges from hundreds to thousands of cases. The number of cells contained in all samples far exceeds the processing capacity of scRNA sequencing. Second, the module clustered by WGCNA is the specific gene set of different cells, which can be calculated only by high-throughput data sampling. However, to date, no study using WGCNA has reached conclusive findings. Most studies have used prognosis and tumor stage (Giulietti et al., 2018; Jiang et al., 2020) or immune cells (Lin et al., 2020) as the relevant phenotype to find the key cancer-related immune genes.

Regarding cancer gene prognosis models, kidney cancer research has been reported, but the genes used to build the models were different (Zhang et al., 2020a,b). This is highly related to the proportion of cells in the detected cancer tissue, so the results will also vary greatly. Relying on gene prognostic models presents very limited options for accurate prognosis. Conversely, assessing the degree of tumor fibroblast infiltration to determine prognosis is more widely applicable and stable. Prognosis by fibroblasts intervention in KIRC patients was confirmed in our study. However, further clinical studies are warranted before this gene signature can be validated as a potential biomarker of CAFs in KIRC.

## Conclusion

In summary, we demonstrated that the increased infiltration of fibroblasts in KIRC is significantly associated with tumor stage, pathological grade, and prognosis. WGCNA is a robust method for identifying the specifically expressed gene signature of CAFs in the complex KIRC TME. Gene signature with clinical significance is an important basis for our future research on KIRC CAFs and lays the foundation for future tumor-targeted CAF treatment.

## Data Availability Statement

Publicly available datasets were analyzed in this study. This data can be found here: The datasets used and/or analyzed during the current study are available from GEO (https://www.ncbi.nlm.nih.gov/geo) and TCGA (https://portal.gdc.cancer.gov/repository).

## Author Contributions

BL proposed the study concept, designed, and drafted the manuscript. BL and SP collected, analyzed, and interpreted the data. YZ, XC, and BW participated in revising the manuscript. All authors read and approved the final manuscript.

## Conflict of Interest

The authors declare that the research was conducted in the absence of any commercial or financial relationships that could be construed as a potential conflict of interest.

## Abbreviations

CAFs: cancer-associated fibroblasts
DEGs: differentially expressed genes
EPIC: Estimate the Proportion of Immune and Cancer cells
ESTIMATE: Estimation of STromal and Immune cells in MAlignant Tumor tissues using Expression data
GEO: Gene Expression Omnibus
GS: gene significance
IHC: immunohistochemistry
KIRC: kidney renal clear cell carcinoma
MM: module membership
RCC: renal cell carcinoma
ROC: receiver operating characteristic
scRNA: single-cell RNA
ssGSEA: single-sample gene set enrichment analysis
TCGA: The Cancer Genome Atlas
TME: tumor microenvironment
TOM: topological overlap matrix
WGCNA: Weighted Gene Coexpression Network Analysis.
